# Bioflavonoid-Induced Apoptosis and DNA Damage in Amastigotes and Promastigotes of *Leishmania donovani*: Deciphering the Mode of Action

**DOI:** 10.3390/molecules26195843

**Published:** 2021-09-27

**Authors:** Shaila Mehwish, Sanjay Varikuti, Mubarak Ali Khan, Tariq Khan, Imdad Ullah Khan, Abhay Satoskar, Hamed Abdelhamid Elsayed Elserehy, Nazif Ullah

**Affiliations:** 1 Department of Biotechnology, Faculty of Chemical and Life Sciences, Abdul Wali Khan University Mardan, Mardan 23200, Pakistan; biotechwish@gmail.com (S.M.); makhan@awkum.edu.pk (M.A.K.); ik16092@gmail.com (I.U.K.); 2 Department of Pathology, The Ohio State University Medical Center, Columbus, OH 43210, USA; sanjay.varikuti@osumc.edu (S.V.); abhay.satoskar@osumc.edu (A.S.); 3 Department of Biotechnology, Faculty of Biological Sciences, University of Malakand, Chakdara 18800, Pakistan; tariqkhan@uom.edu.pk; 4 Department of Zoology, College of Science, King Saud University, Riyad 11451, Saudi Arabia; hel_serehy@yahoo.com

**Keywords:** apoptosis, DNA damage, gallic acid, leishmaniasis, *Leishmania donovani*, quercetin, rutin

## Abstract

Natural products from plants contain many interesting biomolecules. Among them, quercetin (Q), gallic acid (GA), and rutin (R) all have well-reported antileishmanial activity; however, their exact mechanisms of action are still not known. The current study is a step forward towards unveil the possible modes of action of these compounds against *Leishmania donovani* (the causative agent of visceral leishmaniasis). The selected compounds were checked for their mechanisms of action against *L. donovani* using different biological assays including apoptosis and necrosis evaluation, effects on genetic material (DNA), quantitative testing of nitric oxide production, ultrastructural modification via transmission electron microscopy, and real-time PCR analysis. The results confirmed that these compounds are active against *L. donovani*, with IC_50_ values of 84.65 µg/mL, 86 µg/mL, and 98 µg/mL for Q, GA, and R, respectively. These compounds increased nitric oxide production and caused apoptosis and DNA damage, which led to changes in the treated cells’ ultrastructural behavior and finally to the death of *L. donovani*. These compounds also suppressed essential enzymes like trypanothione reductase and trypanothione synthetase, which are critical for leishmanial survival. The selected compounds have high antileishmanial potentials, and thus in-vivo testing and further screening are highly recommended.

## 1. Introduction

Leishmaniasis is a life-threatening disease that causes cutaneous and mucocutaneous ulcers. It is endemic to 98 countries and affects about 350 million people worldwide. The disease is caused by 20 different species of the protozoal parasites leishmania. The death toll from leishmaniasis reaches 70,000 per year, with an incidence of 0.5 million cases of the disease’s visceral form [1]. Visceral leishmaniasis (VL) is the most lethal form of this disease, caused by *Leishmania donovani* and *Leishmania infantum*. It affects the visceral body parts, spleen, liver, and bone marrow and is characterized by weight loss and anemia [2].

Leishmaniasis is a poverty-related disease and compromises the health and wellbeing of people specified in the major sustainable development goals (SDGs). Its treatment is a major issue in developing countries. Currently available leishmaniasis treatments include the pentavalent antimonial, stibogluconate, meglumine antimoniate, liposomal amphotericin B, miltefosine, pentamidine, and paromomycin. People use drugs such as amphotericin B and paromomycin for the treatment of leishmaniasis, which are limited by high cost, resistance issues, toxicity to humans and the environment, and severe pain associated with injection. Drug resistance in Leishmania species and environmental toxicity from high doses of these drugs are serious issue that require urgent attention [3]. Therefore, it is crucial to find more efficient drugs against this form of leishmaniasis [4]. In the past few years, the potential of secondary metabolites of plants as environmentally safe reactive components has become prominent. Plant-derived natural products such as phenolics, flavonoids, alkaloids, and saponins provide environmentally safer and less toxic alternatives to solve problems associated with the current drugs used against leishmaniasis [5].

Flavonoids are plant-derived natural compounds which carry high antileishmanial potential against different forms of leishmaniasis. Flavonoids also offer promise in applications against many diseases including cancer, inflammation, parasitic disorders, and infectious diseases [6]. The mechanism through which flavonoids are thought to produce their strong activity is through their antioxidant potential [7]. Moreover, flavonoids have been shown to modulate reactive oxygen species (ROS)-scavenging enzyme activities, participate in arresting the cell cycle, induce apoptosis and autophagy, and suppress cancer cell proliferation and invasiveness. Flavonoids have dual action regarding ROS homeostasis—they act as antioxidants under normal conditions and are potent pro-oxidants in cancer cells, triggering the apoptotic pathways and downregulating pro-inflammatory signaling pathways [8]. This makes flavonoids the best candidates for the treatment of different ailments, including leishmaniasis. Several studies have already reported the use of flavonoids against Leishmania [9]. However, none of the reported compounds have been developed into a widely used therapeutic agent against leishmaniasis. This is mainly because the existing research on herbal medicine and other natural compounds against leishmaniasis is limited to preliminary testing only [5]. Quercetin (Q), gallic acid (GA), and rutin (R) are among the natural flavonoids previously reported for their antileishmanial activity; however, their mechanism of action is still not known. To establish their efficacy against leishmaniasis, mechanistic studies of such compounds could provide insight into their potential targets, which may lead to the discovery of new drugs in this field [10,11,12].

## 2. Results and Discussion

### 2.1. Effects of Compounds on Viability of Promastigotes

**Figure 1:**
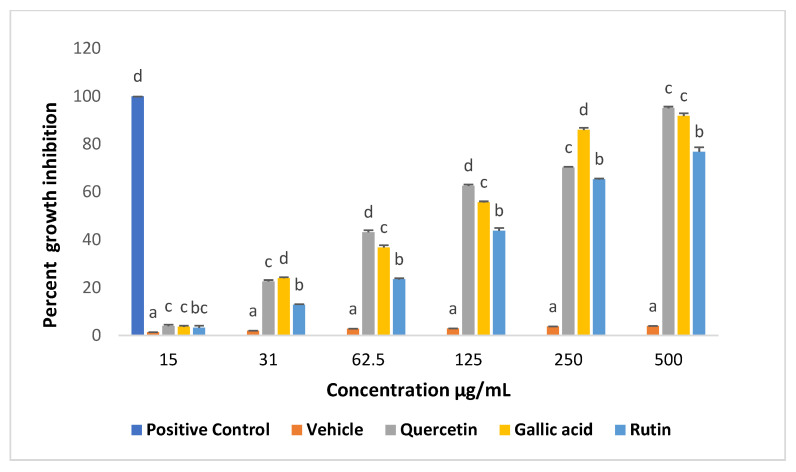
Percent growth inhibition of the Leishmania (Ds-Red *L. donovani* strain LV82) promastigotes caused by quercetin, gallic acid, and rutin at concentration equal to their IC_50_ (µg/mL) values. Data represent the mean values of three replicates with ± standard error. Different labels on columns show significant difference at *p* < 0.05.

In order to investigate the effects of selected compounds on parasite growth and development, promastigotes of *L. donovani* were cultured in the presence and absence of different concentrations (15–500 μg/mL) of Q, GA, and R. Cell density was measured every 24 h for 72 h. A reduction in the growth of promastigotes was observed soon after first 24 h, and increased with the increase in time and concentration of the compounds. Relatively higher growth inhibition activity was observed for Q when compared to GA and R, while an exponential growth was observed in untreated cells. Overall, the data indicated that the selected compounds exhibited significant dose-dependent antileishmanial activity, with IC_50_ values of 84.65 µg/mL, 86 µg/mL, and 98 µg/mL for Q, GA, and rutin, respectively (Figure 1). Quercetin is also known for potent its trypanocidal activity, with an IC_50_ of 8.3 µg/mL [13,14]. Researchers have also explored the promising anticancer potentials of gallic acid and rutin, with IC_50_ values of 12.3 µg/mL and 18.4 µg/respectively. Indeed, it has been reported that rutin inhibits PLA2 activity, an important enzyme in the arachidonic acid cascade, in human synovial fluid [15].

### 2.2. Effects of Compounds on the Viability of Intracellular Amastigotes

**Figure 2:**
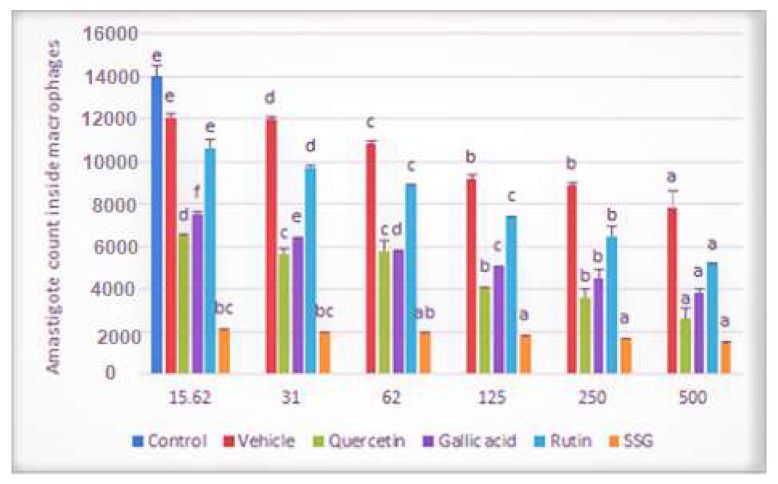
Graph showing amastigote count inside 1000 raw macrophages following treatment with quercetin, gallic acid, and rutin at 15.62 μg/mL–500 μg/mL after 72 h of incubation compared with vehicle control. Data represent the mean values of three replicates with ± standard error. Different labels on column data show significant difference at (*p* < 0.05).

To investigate the effects of the selected compounds (Q, GA, and R) on amastigotes, infected macrophages were treated with increasing concentrations of the selected compounds (15.62 μg/mL–500 μg/mL). With the increases in the selected compounds’ concentrations, co-linear decreases in the number of amastigotes inside macrophages were observed (Figure 2).

**Figure 3:**
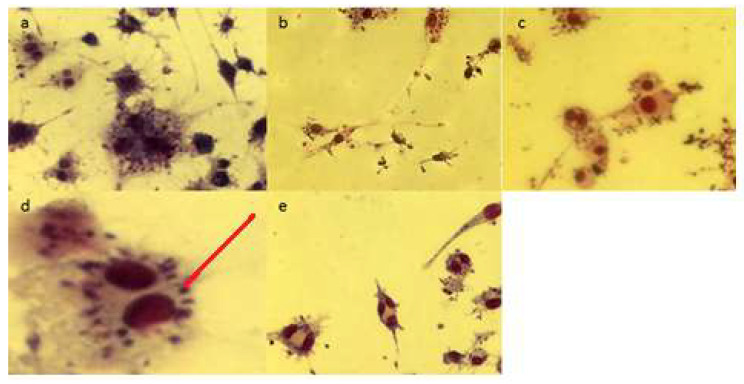
Amastigotes inside macrophages shown with arrows: (**a**) untreated control, (**b**) treated with quercetin, (**c**) treated with gallic acid, and (**d**) treated with rutin at the highest concentration tested (500 μg/mL) after 72 h of incubation, stained with Giemsa stain. (**e**) SSG 250 μg/mL was used as positive control. Images were taken with a Nikon microscope.

Micrographs of Giemsa-stained *L.-donovani*-infected macrophages incubated with quercetin, gallic acid, and rutin are shown in Figure 3.

**Figure 4:**
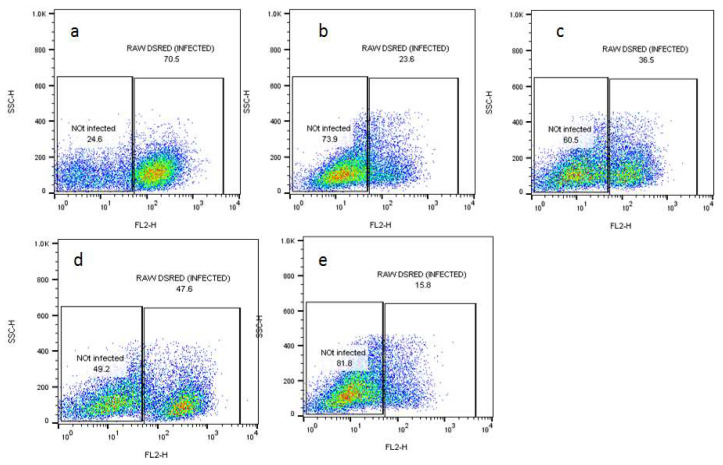
BMDMs infected with transgenic *L. donovani* parasites expressing the red fluorescent protein Ds-Red for the evaluation of parasitic loads using flow cytometry. Treatment was conducted with (**a**) untreated control and cells treated with (**b**) quercetin, (**c**) gallic acid, and (**d**) rutin at their inhibitory concentrations (IC_50_) (84.65 µg/mL, 86 µg/mL, and 98 µg/mL, respectively) for 72 h. (**e**) SSG 250 µg/mL was used as a positive control.

**Figure 5:**
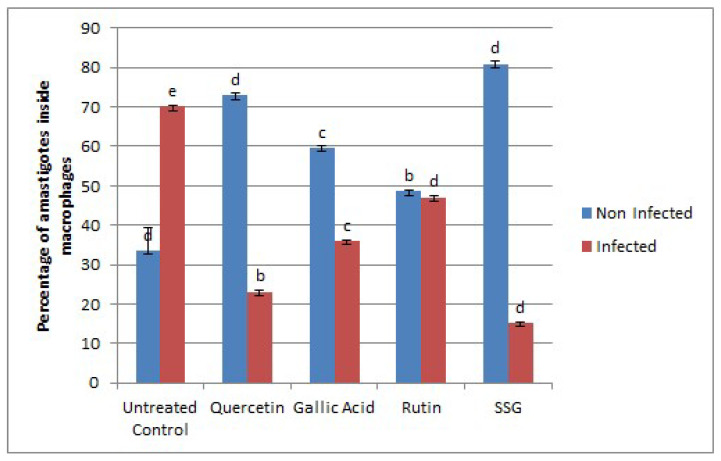
Percentage of amastigotes inside BMDMs infected with transgenic *L. donovani* parasites. Treatment was conducted with quercetin, gallic acid, and rutin at their 50% inhibitory concentrations (IC_50_) (84.65 µg/mL, 86 µg/mL, and 98 µg/mL, respectively) for 72 h. SSG at 250 µg/mL was used as a positive control. Data represent the mean values of three replicates with ± standard error. Different labels on columns show significant difference at *p* < 0.05.

The activity was further confirmed by a flow cytometry assay for which bone-marrow-derived macrophages were infected with Ds-Red *L. donovani* promastigotes that showed fluorescence-emitting amastigotes inside or after bursting from macrophages. To evaluate the transfectant’s infectivity, an in vitro infection study using flow cytometry was used to differentiate live and dead parasites [16]. The assay was conducted for 72 h at the IC_50_ values of the selected compounds for promastigotes, and the absolute fluorescence of infection and the percentage of infected macrophages were measured. Flow cytometry analyses showed that the maximum infection level (70.5%) was achieved within 72 h. Overall, *L. donovani* Ds-Red parasites were found to be fully infective to macrophages. The number of amastigotes decreased from 47.6% (rutin) to 36.5% (gallic acid) and 23.6% (quercetin) (Figure 4 and Figure 5). The results were compared with SSG, which had 15.8% live parasite in the infected portion. The comparatively higher activity of quercetin against both the amastigotes and promastigotes may be due to the ability of quercetin to affect multiple targets at a time, such as DNA, cell membrane, and the expression of enzymes such as those involved in cell cycle arrest and apoptosis [13,17,18,19].

### 2.3. Fluorescence Microscopy Assay for Apoptosis and Necrosis

**Figure 6:**
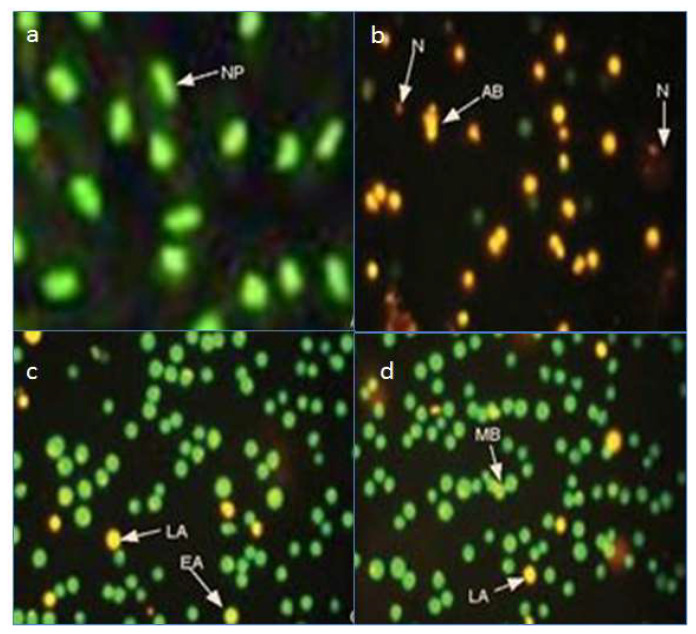
Images taken with fluorescent microscope: (**a**) untreated leishmania promastigotes and treated with (**b**) quercetin, (**c**) gallic acid, and (**d**) rutin. AB: apoptotic body, LA: late apoptosis, EA: early apoptosis, MB: membrane blebbing, N: necrosis, and NP: normal promastigotes.

Apoptosis, the phenomenon of cell death, is a complex phenomenon that involves a series of intercellular events including, but not limited, to membrane permeability, DNA damage, and activation of caspases [3,20]. Caspase activation causes cellular proteins to degrade; as a result, cell shrinkage occurs, which may lead to membrane blebbing, condensation of chromatin, and finally DNA breakage [20]. Amongst the selected compounds, quercetin showed the highest apoptotic activity, followed by gallic acid and rutin. Quercetin-treated cells were mostly apoptotic and necrotic, while gallic acid and rutin caused early and late apoptosis and membrane blebbing [21]. Typical apoptotic and necrotic characteristics such as apoptotic bodies, late apoptosis, early apoptosis, membrane blebbing, and necrosis were observed in the treated cultures, as shown in Figure 6. Early and late apoptosis were characterized by chromatin condensation and orange to red nuclei, respectively, as previously described by [22,23]. Our study confirmed the apoptotic potential of the important bioflavonoids Q, GA, and rutin, among which quercetin has been previously reported to exert caspase-independent apoptotic effects on *Leishmania major* (the causative agent of cutaneous leishmaniasis) promastigotes, and reactivate the death of infected phagocytes [24]. The loss of mitochondrial membrane potential and apoptosis-related death in *L. donovani* has also been reported for other phenolics including rosmarinic acid and apigenin [25].

### 2.4. Effects of Compounds on DNA of Treated Promastigotes

**Figure 7:**
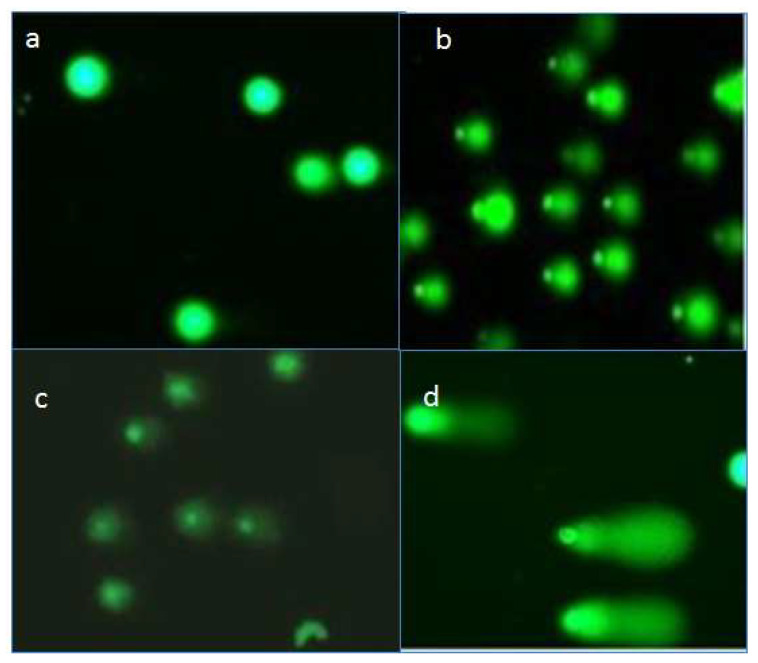
Images of EtBr-stained DNA migration towards anode. Damaged DNA from *L. donovani* was differentiated via alkaline comet assay: (**a**) normal DNA (untreated control) and DNA from samples treated with (**b**) quercetin, (**c**) gallic acid, and (**d**) rutin at (500 μg/mL) after 72 h of incubation.

Small molecules can bind to DNA and thus can cause severe damage that may involves conformational changes in DNA or other physiological changes such as double-stranded breaks. The Comet assay is one of the most sensitive and robust methods to determine damage to and breaks in DNA strands. The results obtained via this assay showed apparent DNA damage in Leishmania, as evidenced by the tails displayed for all three treatment groups compared to the control group (Figure 7).

**Figure 8:**
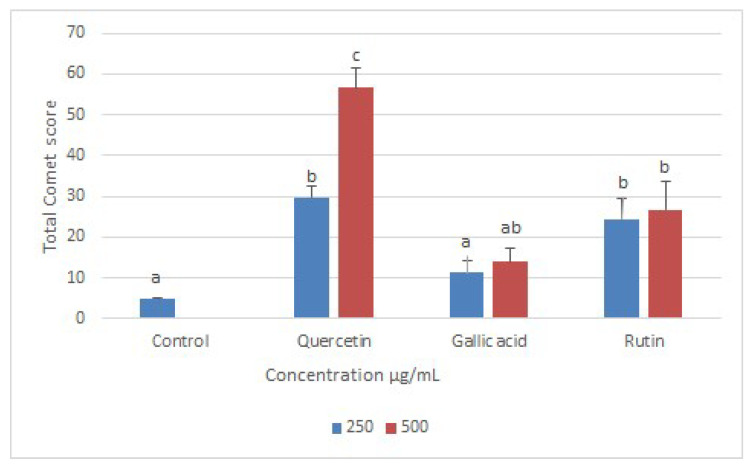
Total comet scores of leishmanial DNA damage following treatment with quercetin, gallic acid, and rutin, compared with control DNA (from untreated Leishmania). Data represent the mean values of three replicates with ± standard error. Different labels on columns show significant difference at *p* < 0.05.

The result showed that quercetin caused most significant DNA damage with a TCS score of 57, followed by rutin (26) and gallic acid (14) at their highest tested concentrations (500 μg/mL) (Figure 8). The DNA-binding abilities of quercetin, GA, and R have been shown; they can bind to DNA directly via intercalation and hence can mediate double-stranded DNA (ds-DNA) damage in situ [26,27,28].

### 2.5. Nitric Oxide (NO) Production

**Figure 9:**
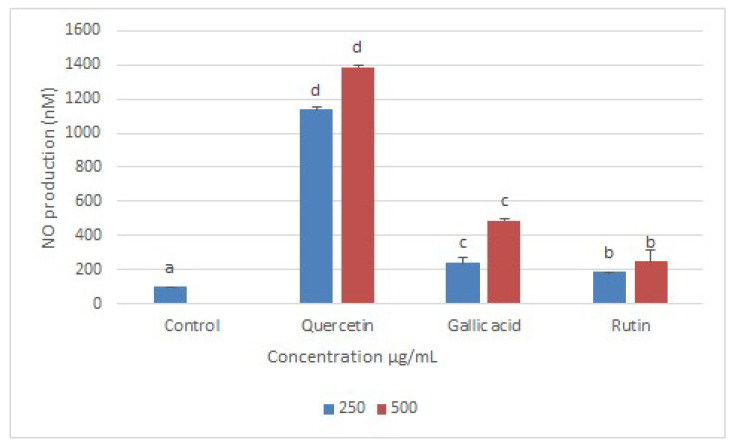
Quantification of NO production by BMDMs infected with *L. donovani* and treated with Q, GA, and R at 250 µg/mL and 500 µg/mL for 72 h. Infected macrophages without treatment served as a control. Data represent the mean values of three replicates with ± standard error. Different labels on columns show significant difference at *p* < 0.05.

It is an established phenomenon that DNA damage can produce reactive oxygen species (ROS), which can consequently cause oxidative outburst in cells [29,30]. Conversely, high levels of ROS can lead to impaired physiological function through cellular damage of DNA, proteins, lipids, and other macromolecules, which can lead to various human pathologies including cancers, neurodegenerative disorders, and cardiovascular disease, as well as aging [31]. Furthermore, an elevated level of NO production is closely related to a high level of oxidative stress, which in turn leads to apoptosis-related cell death [32]. In many studies, leishmanial cell death has been related to the upregulation of NO [33]. In this investigation, a Griess reagent assay was conducted to measure the nitrite (end-product of cellular NO) produced after the treatment of leishmanial cells with Q, GA, and R. The supernatants of Leishmania-infected BMDMs treated with quercetin, gallic acid, and rutin showed significantly higher levels of nitric oxide production compared with untreated cells, with the Q-treated group producing the highest amount of NO, followed by GA and R at 500 µg/mL (Figure 9).

Like many other flavonoids, quercetin is known to interfere with inducible nitric oxide synthase activity [34,35]. The much higher amount of nitric oxide produced by inducible nitric oxide synthase in macrophages can cause oxidative damage to leishmania cells inside macrophages. Meanwhile, macrophages can neutralize the same oxidative damage via other mechanisms [36].

### 2.6. Real-Time PCR Assay

**Figure 10:**
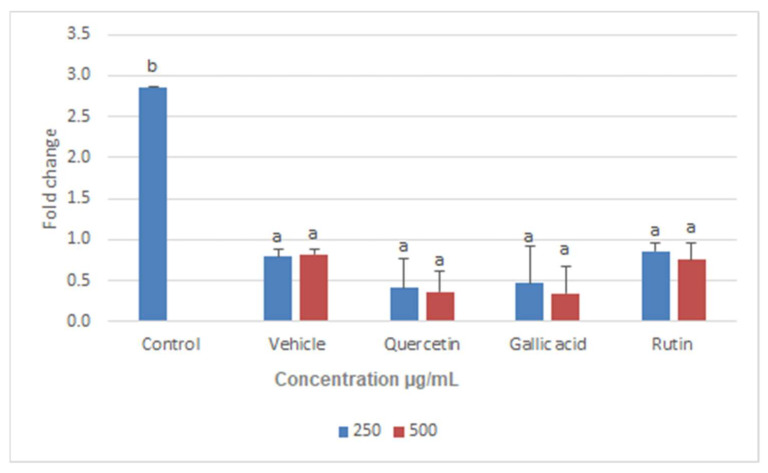
Fold change in expression level of trypanothione reductase enzyme in *Leishmania donovani* after treatment with two different concentrations (250 µg/mL and 500 µg/mL) of quercetin, gallic acid, and rutin. Data represent the mean values of three replicates with ± standard error. Different labels on columns show significant difference at *p* < 0.05.

**Figure 11:**
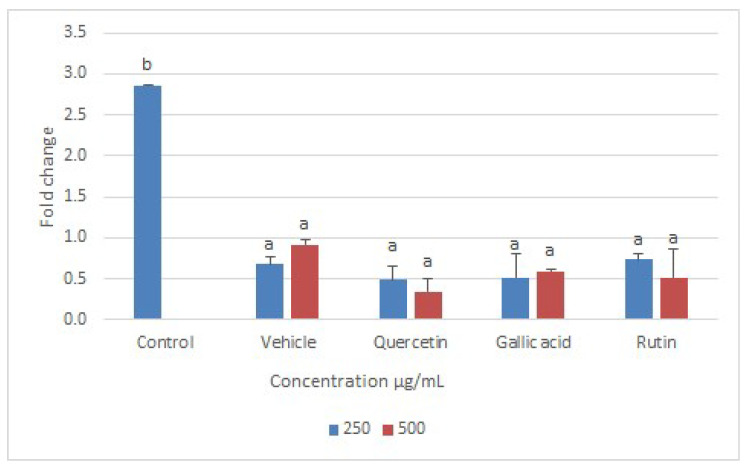
Fold change in expression level of trypanothione synthetase enzyme in *Leishmania donovani* after treatment with two different concentrations (250 µg/mL and 500 µg/mL) of quercetin, gallic acid, and rutin. Data represent the mean values of three replicates with ± standard error. Different labels on columns show significant difference at *p* < 0.05.

Disruption of mitochondria can lead to the disruption of all critical metabolic pathways, including enzymatic functions essential for parasite survival [37]. In the current study, Q, GA, and R were checked for their regulatory effects on two important enzymes, namely trypanathion reductase (Try-R) and trypanathion synthase (Try-S). These enzymes were selected for the expression analysis not only because they are unique to Leishmania (i.e., not present in humans), but also because they play an essential role in the survival of leishmanial bodies [38]. Furthermore, Q, GA, and R showed strong interactions with Try-R and Try-S and hence reduced their activity [38]. Our study confirmed that both the enzymes (Try-R and Try-S) were downregulated when *L. donovani* was treated with the selected compounds Q, GA, and R for 24 h (Figure 10 and Figure 11), and hence lead to the death of leishmania. The lowest expression levels were observed upon treatment with Q, followed by R and GA at 500 µg/mL. A preliminary investigation made in previous studies demonstrated the effects of quercetin on the expression of genes related to cell cycle arrest and apoptosis in human colon-adenocarcinoma cells [19]. The expression levels of enzymes were compared with that of beta-actin (housekeeping gene), and Cq values were measured with targeted genes.

### 2.7. Transmission Electron Microscopy

**Figure 12:**
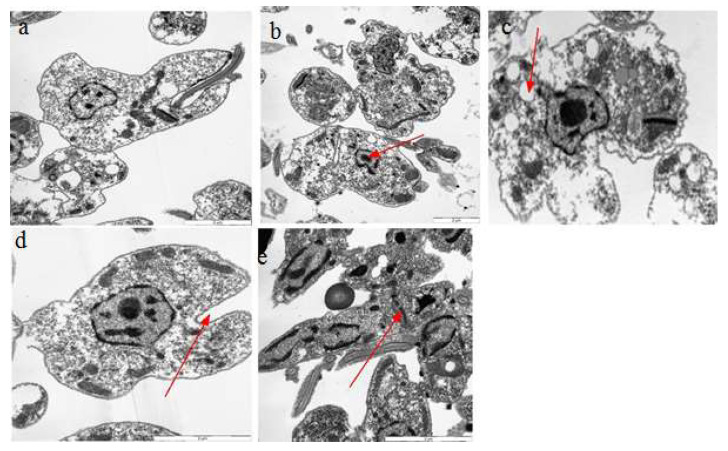
Ultrastructural changes in *L. donovani* LV82 promastigotes treated with quercetin at 500 μg/mL for 72 h. (**a**) Normal cells, (**b**) nuclear condensation, (**c**) appearance of lipid reservoirs, (**d**) distortion of the flagellar pocket, and (**e**) disruption of the mitochondria–kinetoplast complex.

**Figure 13:**
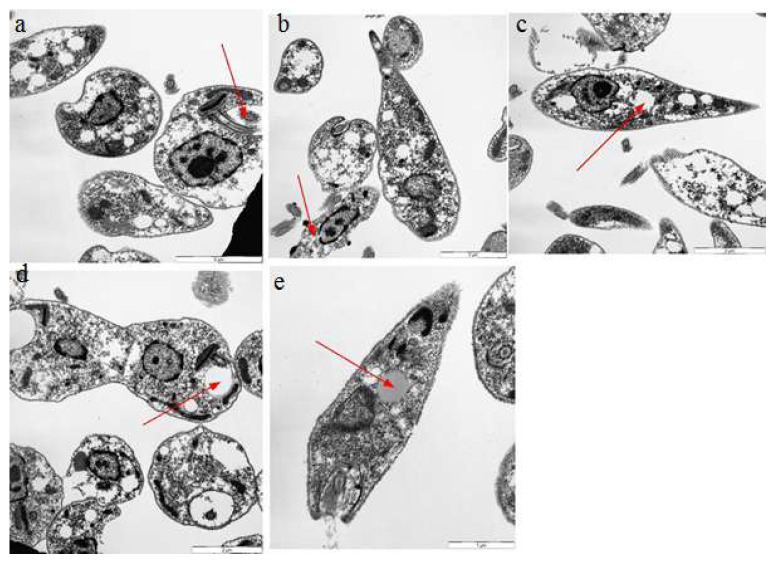
Ultrastructural changes in *L. donovani* LV82 promastigotes treated with gallic acid at 500 μg/mL for 72 h. (**a**) Distortion of the flagellar pocket, (**b**) acidocalcisomes, (**c**,**d**) large number of vacuoles, and (**e**) lipid reservoirs.

**Figure 14:**
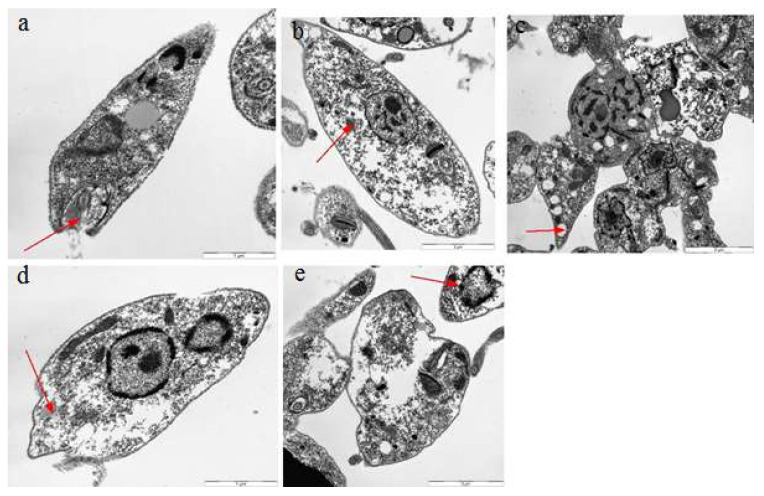
Ultrastructural changes in *L. donovani* LV82 promastigotes treated with rutin at 500 μg/mL for 72 h. (**a**) Distortion of the flagellar pocket, (**b**) acidocalcisomes, (**c**) large number of vacuoles, (**d**) lipid reservoirs, and (**e**) nuclear condensation.

Different bioactive compounds cause DNA damage in cells, producing free radicals and potentially causing ultrastructural changes inside the cell [29]. We exploited the TEM assay to check the ultrastructural changes that occurred in the cell body of Leishmania after treatment with Q, GA, and R. A leishmania cell normally has an elongated kinetoplast with highly condensed DNA, and a single mitochondrion with proper cristae that are extended throughout the parasite cell [39]. Our results confirmed radical changes in the ultrastructures of leishmania cells when exposed to quercetin, gallic acid, and rutin. Changes like nuclear condensation, distortion of the flagellar pocket, disruption of the mitochondria–kinetoplast complex, and increase in lipid droplets were noticeable when observed under TEM. Such changes have been previously reported in *L. amazonensis* when treated with squalene synthase inhibitors and amiodarone. [25]. Evidence of ultrastructural changes is shown in Figure 12, Figure 13 and Figure 14. The results showed that rutin caused more ultrastructural changes in the treated cells compared to quercetin and gallic acid. It was also observed that ultrastructural changes increased with increasing concentrations of the compounds. TEM experiments play an important role in determining the mechanisms of action of target compounds. They were previously used by Fernando Almeida-Souza, et al. (2018) to find the mechanism of action of *Morinda-citrifolia*-induced death in *L. infantum* promastigotes [40]. The presence of many large vacuoles (Figure 13) can be correlated with the entry of substances into vacuoles via simple diffusion which occurs due to membrane permeability, as previously shown by [41]. Moreover, the presence of vesicles may represent autophagosomes, which indicates the process of autophagy. Autophagy is a protective mechanism to remove damaged organelles; however, its excess may cause cellular death [42].

The large number of lipid reservoirs indicated the production of abnormal lipids, which accumulate in the cell as a result of drug action or may represent diminished levels of necessary proteins produced by cells [43]. Abnormal lipid production can distort the flagellar pockets, as observed in the treated promastigotes. This indicates the intense exocytic activity of the test compounds. Such changes were previously reported in *L. amazonensis* treated with ergosterol synthesis inhibitors (22,26-azasterol) [44].

## 3. Materials and Methods

Major equipment used in this study were available at Ohio State University, Columbus, Ohio. Saponin, sodium stibogluconate (SSG), and primers were purchased from integrated DNA technology (IDT Coralville, IA, USA); SYBR green and cDNA KIT were purchased from Bio-Rad (Hercules, CA, USA). Reagent-grade potassium chloride, sodium bicarbonate, N-hexane, ethyl acetate, chloroform, dimethyl sulfoxide (DMSO), acridine orange (AO), Hank’s balanced salt solution, hydrogen peroxide (H_2_O_2_), RPMI1640, fetal bovine serum (FBS), penicillin–streptomycin solution, Triton X-100, and the flavonoids quercetin, gallic acid, and rutin were purchased from Sigma-Aldrich (St. Louis, MO, USA). Double-distilled deionized water was used in all experiments.

### 3.1. Parasites and Cell Cultures

Ds-Red *L. donovani* (biochemically engineered *Leishmania donovani* LV82 strain), which expresses a red florescent protein, was cultured in RPMI-1640 medium supplemented with 10% fetal bovine serum (FBS) and 1% penicillin and streptomycin solution at 25 ± 1 °C [45]. Bone-marrow-derived macrophages (BMDMs) were acquired according to a previously published protocol [46]. The use of murine BMDMs was in accordance with the approved protocol (2010A00048-R3) by OSU-Institutional Animal Care and Use Committee). Briefly, tibia and femur bones of BALB/c mice were separated, and bone marrow was flushed with PBS using fresh syringes. When recovering the bone marrow of all bones, centrifugation of tubes containing the cells was employed at 1500 rpm, for 7 min at 4 °C. Cells were recovered and plated at 1 × 10^6^ /mL (RPMI-1640 medium containing 10% (v/v) L929 cell supernatant, 10% fetal bovine serum (FBS), 2 mM L-glutamine, 50 µM 2-mercaptoethanol, and 1× penicillin–streptomycin) in 75 cm flasks. Subsequently, six days after plating, nonadherent cells were discarded and adherent macrophages were scraped from the flasks and plated at 0.5 × 10^6^ /mL in 24-well plates.

### 3.2. Effect of Compounds on Viability of Promastigotes

The effects of flavonoids on the viability of promastigotes was assessed by treating the promastigote form of *L. donovani* Ds-Red (1 × 10^6^ cells/mL) with six different concentrations of selected compounds (Q, GA, and R), i.e., at 15–500 µg/mL. After 72 h, 10% Alamar blue dye was added to all wells followed by incubation for 6 h at 26 °C. We recorded the spectrophotometric readings at 570 nm. The percent viability was derived using the following formula:

Percent Viability = (O.D. of Sample-O.D. of negative control)/(O.D. of Blank-O.D. of negative control) × 100)

where O.D. means optical density.

Saponin was used as a positive control. The experiments were performed in triplicate.

### 3.3. Effects of Compounds on Viability of Intracellular Amastigotes (via Giemsa Stain)

Adhered raw macrophages were infected with *L. donovani* promastigotes and cultures were treated with different concentrations of the selected compounds for 72 h [12]. Afterwards, cells were washed with warm phosphate buffer saline (PBS) and fixed with 300 µL ice-cold methanol. After 10 min, methanol was removed from the wells and cells were dried and further stained with Giemsa for 30 min. Excess stain was removed by washing thoroughly with distilled water. The number of parasites inside the macrophages was determined by examination under a microscope. Numbers of amastigotes were determined by counting 1000 nucleated cells. SSG was used as a positive control and the experiments were performed in triplicate.

### 3.4. Flow Cytometry for Assessing the Effects of Compounds on Viability of Intracellular Amastigotes

The bone-marrow-derived macrophages were plated until adherence to the surface in a 24-well plate. The adhered macrophages were then infected with DS-Red *L. donovani* parasite for 24 h at a ratio of 1:10 (macrophages:parasites) and washed twice with warm PBS, followed by the addition of different inhibitory concentrations (IC_50_) (84.65 µg/mL, 86 µg/mL, and 98 µg/mL) of the selected compounds and incubation for 72 h. The infected macrophages were centrifuged in FACS tubes at 1250 rpm for 5 min and resuspended in 300 µL PBS buffer and the parasitic loads inside the macrophages were recorded using a flow cytometer. Parasitic loads were determined by gating on the cells with Flow Jo software [47]. Sodium stibogluconate (SSG) was used as a positive control.

### 3.5. Fluorescence Microscopy Assay for Apoptosis and Necrosis

Apoptosis and necrosis were determined according to the method described in [48]. The drug-treated parasites were stained with ethidium bromide and acridine orange (3:1). The variance in fluorescence was measured using a Leica fluorescent microscope with a Canon camera using 530 and 485 nm filters for emission and excitation wavelengths, respectively. Triton X-100 (0.5%) was used as a positive control.

### 3.6. Assessment of the Effect of Compounds on DNA of Promastigotes

The DNA damage assay, i.e., comet assay, was performed according to the method described by [30]. The Leishmania cells were mixed with low-melting-temperature agarose and put onto the slides. After solidifying, the slides were subjected to gel electrophoresis at 24 volts for 30 min. The slides were then taken out and washed with neutralization buffer for 5 min followed by ethanol for drying. The treated DNA was stained with ethidium bromide and was observed under a fluorescent microscope. Treated cells were counted to score comets by a simple method described by Collins [49]. For this purpose, 100 cells were randomly selected for the damage DNA score. The total comet score was then calculated using the following formula:

Total Comet Score (TCS) = 0(n.) + 1(n.) + 2(n.) + 3(n.) + 4(n.)

The Leishmania-mediated killing could be related to the upregulation of NO by BMDMs [50]. A Griess assay was conducted in order to measure the level of nitrite. Nitrite is the end product of cellular NO, produced after treatment with two different concentrations of the selected compounds (250 µg/mL and 500 µg/mL) for 72 h. A stock solution of 10 mM sodium nitrite (NaNO_2_) was made. A serial dilution was started with 40 µM in the first well, followed by serial dilution with RPMI such that each well in the 96-well plates had 50 µL of reagent standard. Subsequently, 50 µL of sample (supernatant) was poured into each well followed by the addition of 50 µL of Griess reagent for standard and samples, keeping 2–3 wells blank (with RPMI media and Griess reagent). The readings were taken at 570 nm in ELISA.

### 3.7. Transmission Electron Microscopy for Ultrastructure Analysis

To check the ultrastructural behavior of *L. donovani* LV82, cells were treated with the selected compounds for 72 h [51]. The treated cells were washed two times with PBS, fixed, and observed under transmission electron microscopy (TEM) (JEOL J1010 USA). The untreated parasite cells were used as a control.

### 3.8. Real-Time PCR Assay for Quantification of mRNA

This assay was performed after 24 h treatment of the parasite with the selected compounds. According to the manufacturer’s protocol, total RNA from the treated cells was extracted from *L. donovani* Ds-Red using the Qiagen RNase isolation kit.

Reverse transcription was performed using the Superscript III reverse transcriptase kit (Life Technologies, Grand Islands, NY, USA). The synthesized cDNA was amplified for trypanothione reductase (Try-R) and trypanothione synthetase (Try-S) enzymes. Real-time PCR was performed in the Step One Plus TM system (Applied Biosystems) using SYBR Green (Invitrogen) chemistry. The primers used for trypanothione reductase were GCTGCGTGCCAAAGAAACTC (F) and GAAAGCTGAGGCCCTCCG (R), and those for trypanothione synthetase were CTGAGTCTGGTGGCAACTATGG (F) and GTTTGTCGGTCTTGATGCGA (R). The primers were designed by Primer Express (Applied Biosystems, Foster City, CA, USA). Following the initial denaturation step at 95 °C for 10 min, target genes were amplified by 40 cycles of denaturation at 95 °C by annealing and extension at 60 °C for 1 min. The fold change in the expression levels was determined via the ∆∆Cq method [52].

### 3.9. Statistical Analysis

All the results were generated using two-way analysis of variance (ANOVA) to compare the results of different treatments with positive controls. The results were further analyzed with Student’s t-test with level of significance *p* < 0.05. All the results are presented as mean ± standard deviation (SD).

## 4. Conclusions

In conclusion, the selected compounds (Q, GA, and R) showed promising antileishmanial activity. These compounds bind to DNA via intercalation, causing DNA damage which may lead to apoptosis and increased nitric oxide production, which in turn create changes in the ultrastructural behavior of *L. donovani* such as nuclear condensation, the appearance of lipid reservoirs, distortion of the flagellar pocket, and the disruption of the mitochondria–kinetoplast complex and finally lead to the death of treated cells.

## Data Availability

Not applicable.

## Sample Availability

Samples of the compounds are not available from authors.
